# Cognitive training and neuromodulation for Alzheimer treatment

**DOI:** 10.18632/aging.204044

**Published:** 2022-04-27

**Authors:** Fabrizio Vecchio

**Affiliations:** 1 Brain Connectivity Laboratory, Department of Neuroscience and Neurorehabilitation, IRCCS San Raffaele Roma, Rome, Italy; 2 Department of Theoretical and Applied Sciences, eCampus University, Novedrate (Como), Rome, Italy

**Keywords:** EEG, Small World, cognitive training, rTMS, Alzheimer

Neurodegenerative diseases comprise a wide range of diseases that share the common characteristic of progressive loss of structure or function of neurons and glial cells in the brain and spinal cord. Neurons in neurodegenerative diseases are affected by neuronal dysfunction at the level of synaptic transmission, synaptic contacts, axonal and dendritic degeneration. While the causes associated with neuronal degeneration remain poorly understood, the incidence of neurodegeneration increases with age, in mid−to−late adult life. The term degenerative diseases of the nervous system mainly refers to *dementias*, in relation to the higher prevalence that characterizes them. As a matter of fact, the world’s population is ageing: improvements in health care in the past century have contributed to people living longer and healthier lives. However, this has also resulted in an increase in the number of people with non-communicable diseases, including dementia. Symptoms of dementia are gradual, persistent and progressive. Individuals suffering from dementia change in cognition, function and behavior. The clinical presentation of dementia varies greatly among individuals, and the cognitive deficits it causes can present as memory loss, communication and language impairments, agnosia (inability to recognize objects), apraxia (inability to perform previously learned tasks) and impaired executive function (reasoning, judgement and planning). Alzheimer’s disease (AD) is the most common neurodegenerative disorder in elderly population and the main cause of cognitive impairment.

Current pharmacological therapies for AD do not change its course and do not always have benefits in the patient's state. Therefore, the optimization of quality of life represents the best possible outcome achievable in all stages of the disease. Cognitive and behavioral rehabilitation represents the main therapeutic approach for this purpose, also in order to mitigate the distress of family and caregivers. In recent years, some non-invasive and rehabilitative therapies have been taking place to increase the patient's cognitive well-being.

Several studies in recent years have verified the potential effects of cognitive treatments, such as cognitive stimulation (CS), cognitive training (COG) and cognitive rehabilitation (CR), on brain plasticity with the focused goal of improving brain function.

Cognitive stimulation (CS) is a non-specific approach with a range of different activities and emphasis to social interaction. It originates from both Reality Orientation Therapy [1], that uses reminders about information such as day, time, location, and Reminiscence Therapy [2], that involves the discussion of past experiences with the aid of photographs, household items, music recordings, or other familiar items from the past.

Cognitive training (COG) includes rehabilitation sessions composed of tasks designed to involve cognitive abilities such as memory, attention and language [3]. Tasks may be presented in paper-and-pencil or in computerized form and in the latter one the tasks can be adapted to individual performance.

Cognitive Rehabilitation (CR) in particular aims to develop personalized strategies to deal with cognitive impairments [4]. It is characterized by an individualized approach that identifies goals that are relevant to the patients and their everyday life.

Together with cognitive treatments one of the possible innovative strategies to be undertaken is the neuromodulation that involves non-invasive brain stimulation techniques (NIBS) such as transcranial magnetic stimulation (TMS), transcranial direct current stimulation (tDCS) and transcranial alternating current stimulation (tACS).

Neuromodulation techniques are having a growing consensus as a therapeutic approach of incipient and mild to moderate dementia because of their capability to be modulated both in space, i.e. in different cortical and subcortical areas of the brain, and time.

Based on these assumptions and promising results, particularly of rTMS and COG, some researchers hypothesized that a treatment combining rTMS and COG may result in synergic effects more effective respect to applying the two therapies separately. Recent studies have investigated the effect of this dual stimulation of rTMS-COG in order to evaluate possible improvements and long-term effects. In particular, a recent study [5] analyzed neuropsychological and neurophysiological data, derived from electroencephalography (EEG), to evaluate the effects of a rTMS-COG treatment in Alzheimer patients. The study was designed a randomized, double-blind, sham-controlled trial to evaluate efficacy of rTMS on 6 brain regions obtained by an individual MRI combined with COG related to brain areas to stimulate (i.e. syntax and grammar tasks, comprehension of lexical meaning and categorization tasks, action naming, object naming, spatial memory, spatial attention). Patients underwent neuropsychological and EEG examination before, after treatment and after 40-weeks, to evaluate the effects of rehabilitation therapy. “Small World” (SW) graph approach was introduced to model the architecture of brain connectivity in order to correlate it with cognitive improvements [6–8]. Following 6-weeks of intensive daily treatment the immediate results showed an improvement in cognitive scales among patients. SW present no differences before and after the treatment, whereas a crucial SW modulation emerges at 40-weeks follow-up, emphasizing the importance of rTMS-COG rehabilitation treatment. Additional results demonstrated that the delta and alpha 1 SW seem to be diagnostic biomarkers of AD, whereas alpha 2 SW might represent a prognostic biomarker of cognitive recovery. Derived EEG parameters can be awarded the role of diagnostic and predictive biomarkers of AD progression and rTMS-COG can be regarded as a potentially useful treatment for AD.

In conclusion, rTMS combined with cognitive training, can be regarded as a potentially useful treatment for AD, not modifying the neuropathological changes, but slowing down their effects on brain networks and providing important groundwork for future studies to build upon. Derived EEG parameters can be awarded the role of diagnostic and predictive biomarkers of AD progression.
